# Editorial: Variables involved in promoting psychological wellbeing and positive development during the life cycle

**DOI:** 10.3389/fpubh.2023.1225526

**Published:** 2023-06-07

**Authors:** María del Mar Molero Jurado, África Martos Martínez, María del Carmen Pérez-Fuentes

**Affiliations:** Department of Psychology, Faculty of Psychology, University of Almería, Almería, Spain

**Keywords:** psychological wellbeing, life cycle, positive development, psychological resources, positive adjustment

The COVID-19 pandemic has had a profoundly severe impact on the public's psychological wellbeing. The high rate of infection and death, the collapse of hospitals, high initial uncertainty, the many days of confinement, business closings, and sudden adaptation to home working and online education, are only a few of the things that arose from the pandemic, and which in one way or another reached the public of all ages around the world (1–5). Life suddenly changed, involving a huge effort to adapt and compromising mental health (6, 7).

Although the pandemic caused an enormous worldwide public psychological impact, it also led to a change in the viewpoint of health and mental wellbeing (8). Thus, the need for attention to and taking advantage of the human being's positive inner qualities was accentuated. That is, it was understood that it was absolutely necessary for our society to promote mental health with strategies concentrating on individual capabilities and strengths, thereby favoring those psychological resources and strategies that enable us to cope with adverse situations that threaten psychological wellbeing and prevent the appearance of psychological symptoms.

Therefore, there is no doubt that a special issue in *Frontiers in Public Health* devoted to the exploration of variables promoting psychological wellbeing and positive adjustment is more than justified. In the special issue, “Variables involved in promoting psychological wellbeing and positive development during the life cycle,” we explore important subjects concerning the positive factors that orient individuals toward wellbeing and integral development in any stage of life. The articles published are contributions examining those abilities, skills, aptitudes, and experiences that are positively directed toward the achievement of individual wellbeing and integral development in different populations and stages of the life cycle. Some of the most important contributions presented in this special issue are mentioned below:

Chen et al. estimate a moderated mediation model to find out whether a sense of life mediates the relationship between core self-evaluation and subjective wellbeing, and whether this mediation, in turn, is moderated by self-esteem. In a sample of 1,185 adolescents, they find that core self-evaluation contributed to the subjective wellbeing of youths and that a sense of life mediated the relationship between these variables. Furthermore, self-esteem had a moderating effect, where adolescents with medium or low self-esteem were those who experienced the indirect effect of core self-evaluation in subjective wellbeing through a sense of life. Based on these findings, the authors suggest the possibility of promoting subjective wellbeing through interventions focusing on improving core self-evaluation or working on understanding a sense of life, especially with young people with low self-esteem (Chen et al.).

In a cross-sectional study, Schnettler et al. examine the effects of job satisfaction, family life, and satisfaction related to feeding a parent, on satisfaction with one's own life, that of one's partner, and of adolescent children. The authors find that satisfaction with the life of their parents (both fathers and mothers) was positively associated with satisfaction with their own job, family, and food, and negatively with satisfaction with adolescent children's eating habits. Furthermore, mothers' satisfaction with life was positively associated with fathers' and adolescents' family satisfaction. The authors also study the influence of satisfaction with family life and satisfaction related to adolescent eating, and how these variables influence satisfaction with their own lives and those of their parents. The findings showed that satisfaction with their children's lives was positively related to their own family satisfaction and eating habits, as well as with their parents' job satisfaction, and negatively in the case of satisfaction with life related to parents' eating habits. In view of the results, Schnettler et al. suggest that to improve satisfaction with life, intervention in families with adolescent children should be directed at all its members.

Granados et al. evaluate a group of prisoners at a penitentiary to check the effectiveness of a program for developing socioemotional competencies and self-esteem. After implementing the program, they find that the prisoners who received the intervention showed an increase in positive social behavior, emotional competencies, and self-esteem. The authors, therefore, suggest the importance of developing and implementing psychoeducational programs to improve emotional and social competencies and self-esteem in populations deprived of freedom, as they develop their socioemotional development, and therefore, their reinsertion into society (Granados et al.).

Han et al. study social activity in Chinese older adults and its relationship to depressive symptoms. The authors find a significant negative association between depressive symptoms in older adults in urban environments and activities such as helping others, practicing sports, or using the internet, and with activities such as interacting with friends and using the internet in rural participants. On this basis, they emphasize the need to promote optimal aging by stimulating specific social activities in older adult populations (Han et al.).

In conclusion, this Research Topic provides a general view of positive factors and competencies impacting the wellbeing of different populations. It also makes contributions of interest to the scientific community and positive psychology with the publication of such high-quality articles.

## Author contributions

All authors listed have made a substantial, direct, and intellectual contribution to the work and approved it for publication.
